# Subjects Conceived through Assisted Reproductive Technologies Display Normal Arterial Stiffness

**DOI:** 10.3390/diagnostics12112763

**Published:** 2022-11-11

**Authors:** Magdalena Langer, Pengzhu Li, Theresa Vilsmaier, Marie Kramer, Franziska Sciuk, Brenda Kolbinger, André Jakob, Nina Rogenhofer, Nikolaus Alexander Haas, Robert Dalla-Pozza, Christian Thaler, Felix Sebastian Oberhoffer

**Affiliations:** 1 Division of Pediatric Cardiology and Intensive Care, University Hospital, LMU Munich, 81377 Munich, Bavaria, Germany; 2 Division of Gynecological Endocrinology and Reproductive Medicine, Department of Obstetrics and Gynecology, University Hospital, LMU Munich, 81377 Munich, Bavaria, Germany

**Keywords:** assisted reproductive technologies, abdominal aorta, common carotid artery, two-dimensional speckle tracking, arterial stiffness, peak circumferential strain, pulse wave velocity

## Abstract

Multiple studies reported signs of vascular dysfunction in subjects conceived through assisted reproductive technologies (ART). The assessment of arterial stiffness in this cohort seems beneficial for risk stratification. Regional arterial stiffness of the abdominal aorta (AAO) and the common carotid arteries (CCA) was evaluated sonographically using two-dimensional speckle tracking in subjects conceived through ART and spontaneously conceived peers. Global arterial stiffness was assessed utilizing an oscillometric blood pressure device. The cohorts of 67 ART subjects and 86 spontaneously conceived peers (11.31 (8.10–18.20) years vs. 11.85 (8.72–18.27) years, *p* = 0.43) did not differ significantly in parameters of regional and global arterial stiffness. In the sub-analysis of study participants ≥10 years of age, markers of arterial stiffness did not display significant differences between both groups. However, a higher tendency of brachial systolic blood pressure was demonstrated in the ART cohort compared to the control group (120.18 ± 9.57 mmHg vs. 116.55 ± 8.05 mmHg, *p* = 0.050). The present study displayed no significant differences in arterial stiffness between ART subjects and spontaneously conceived peers. Moreover, this study suggests that arterial stiffness does not elevate more profoundly in ART subjects with increasing age. Further studies are required for a more detailed cardiovascular risk stratification of the ART cohort.

## 1. Introduction

Arterial stiffness plays an important role in the pathophysiology of cardiovascular disease (CVD) and is involved in the onset of coronary heart disease, heart failure and stroke [1,2,3,4,5]. Increased arterial stiffness is considered to be an independent cardiovascular risk factor leading to elevated morbidity and mortality [5,6].

Arterial stiffening is characterized by increased functional rigidity and reduced elasticity of the vessel’s walls [4]. This degenerative process can be induced by age or through atherosclerosis [4,7]. The stiffening of the arteries leads to an augmentation of blood pressure, left ventricular afterload and a decrease in coronary perfusion [2,8].

Assisted reproductive technologies (ART) are defined as fertility-related treatments in which eggs or embryos are handled [9]. ART encompasses procedures such as in vitro fertilization (IVF), intracytoplasmic sperm injection (ICSI), or gamete intrafallopian transfer (GIFT) [9]. More than ten million children worldwide and about three percent of children in Germany have been conceived after using ART [10,11]. According to the “developmental origins of adult disease” hypothesis, the offspring is at higher risk to develop a non-communicable disease such as CVD after being exposed to adverse environmental influences (e.g., maternal obesity, advanced maternal age, gestational weight gain, pre-existing maternal CVD) [12]. During the pregnancy itself, an increased maternal CVD risk might be present if hypertensive disorders of pregnancy or gestational diabetes are present [13]. Therefore, cardiovascular risk factors of parents need to be considered, as they can negatively influence the future offspring’s cardiovascular health [14].

There is contradictory evidence on cardiovascular health of ART subjects. Several studies consider early vascular alterations in subjects conceived after using ART, potentially leading to increased cardiovascular morbidity and mortality later in life, as possible long-term health consequences [15,16,17]. A meta-analysis conducted by Guo et al. revealed a significantly increased blood pressure, suboptimal cardiac diastolic function and elevated vessel thickness as a conspicuous cardiovascular risk profile of the ART offspring [18]. In contrast, Halliday et al. could not demonstrate an elevated cardiometabolic risk in a cohort of 193 ART adults aged 22 to 35 years [19]. Consequently, the cardiovascular morbidity of the ART offspring remains unclear and requires further research.

As CVD counts as one of the leading causes of death by non-communicable diseases worldwide [20], the early detection of subtle vascular alterations can be regarded as crucial to improve the overall patient outcome. In the past, multiple methodologies were introduced to assess arterial stiffness non-invasively [2]. Recently, two-dimensional speckle tracking (2DST) has been established as a novel method to evaluate regional arterial stiffness sonographically [21]. Interestingly, in subjects with and without hypertension, 2DST appeared to be superior to conventional measures and showed a better reproducibility [21,22].

To the best of our knowledge, 2DST has not been applied yet to assess arterial stiffness in ART offspring. Hence, this study aimed to investigate whether subtle changes in regional arterial stiffness can be visualized by 2DST of the abdominal aorta (AAO) and the common carotid arteries (CCA) in ART subjects compared to spontaneously conceived peers. Moreover, pulse wave velocity (PWV, m/s), a marker of global arterial stiffness, was assessed in all study participants via an oscillometric blood pressure device.

## 2. Materials and Methods

### 2.1. Ethical Statement

The study was performed in accordance with the ethical standards of the Declaration of Helsinki. The Ethics Committee of the Medical Faculty of the Ludwig Maximilians University Munich (Munich, Germany) approved this study (protocol code: 20-0844, date of approval: 27 December 2020). All participants gave their written informed consent to participate in the present study. For minor study participants, prior written informed consent was additionally given from parents or legal guardians.

### 2.2. Study Design and Study Population

A cohort of 156 study participants were recruited from the Munich heARTerY-study (Assisted Reproductive Technologies and their effect on heart and arterial function in Youth) between May 2021 and March 2022. The cohort consisted of 70 subjects who were conceived after using assisted reproductive technologies and 86 spontaneously conceived peers. To additionally assess the influence of age on the cardiovascular parameters studied, subjects of different ages (children, adolescents, young adults) were included. ART subjects were recruited in cooperation with the Division of Gynecological Endocrinology and Reproductive Medicine, Department of Obstetrics and Gynecology, University Hospital, LMU Munich. Healthy age- and gender-matched controls without known cardiovascular conditions, who were conceived spontaneously, were acquired through public calls within the greater Munich area.

### 2.3. Assessment of Anthropometric Variables

Bodyweight (kg), body height (cm) and body mass index (BMI, kg/m^2^) were determined. Weight classifications were defined in minors as follows: underweight if <10 percentile (P.), normal weight if ≥10 P. but <90 P., overweight if ≥90 P., obese if ≥97 P. [23]. Weight classifications for participants ≥ 18 years of age were defined as: underweight if BMI < 18.5 kg/m^2^, normal weight if BMI ≥ 18.5 kg/m^2^ but < 25 kg/m^2^, overweight if BMI ≥ 25 kg/m^2^ but <30 kg/m^2^, obese if BMI ≥ 30 kg/m^2^. 

### 2.4. Medical History, Course of Pregnancy and Birth, Maternal Educational Level, Physical Examination

Pre-existing health conditions, smoking status and the regular use of medication was evaluated. Data on pregnancy and birth was assessed retrospectively by screening clinical records and by questioning both parents. The following parameters were included in the final analysis: birth weight (g), birth height (cm), gestational age (week), case of multiple pregnancy, maternal age at birth (years), maternal BMI at conception (kg/m^2^), presence of gestational diabetes and maternal blood pressure during pregnancy ≥ 140/90 mmHg. In addition, maternal educational level was assessed according to the German educational system: no school leaving qualification (0), lower secondary school leaving certificate (1), intermediate secondary school leaving certificate (2), general qualification for university entrance (3), completed apprenticeship (4), completed university degree (5). Further, a physical examination was executed in all study participants.

### 2.5. Assessment of Diet Quality

High adherence to the Mediterranean diet is associated with a positive effect on cardiovascular health [24,25]. In this study, the validated 14-item Mediterranean diet assessment tool by Martínez-González et al. was applied for adult participants [24]. For participants < 18 years, the KIDMED index by Serra-Majem et al. was used [26]. Both questionnaires were translated into German. A score ≥ 8, was considered as high adherence to the Mediterranean diet [24,26].

### 2.6. Vascular Assessment

#### 2.6.1. Ambulatory Blood Pressure Measurement

Brachial systolic blood pressure (SBP, mmHg), brachial diastolic blood pressure (DBP, mmHg) and pulse wave velocity (PWV, m/s) were assessed using an oscillometric blood pressure device (Mobil-O-Graph^®^, IEM GmbH, Aachen, Germany). Oscillometric assessed PWV by the Mobil-O-Graph^®^ displayed acceptable accuracy compared to intra-aortic readings in adult patients [27]. Moreover, pediatric reference values were established in the past [28]. Cuff sizes were selected in accordance with right upper arm circumference. Study participants had to remain in a supine and calm position for at least five minutes before measurement. In accordance with the recommendations of the American College of Cardiology and American Heart Association, three consecutive ambulatory measurement were performed and averaged to enhance data validity [29].

#### 2.6.2. Peak Circumferential Strain, Peak Strain Rate and Arterial Distensibility of the Abdominal Aorta and the Common Carotid Arteries

Sonography of the abdominal aorta (AAO) and both common carotid arteries (CCA) was performed by one investigator for all study participants. A Philips iE33 xMatrix or a Philips Epiq 7G ultrasound device (Philips Healthcare, Amsterdam, The Netherlands) was used for sonographic examination. The AAO was evaluated using either a 3–8 MHz sector or a 1–5 MHz sector array transducer in short subxiphoid view at epigastric level. Both CCA were recorded in a supine position. The neck was extended up to a 45° angle and turned to the opposite side of examination. The area right below the carotid bifurcation was examined using a 3–8 MHz sector array transducer in short axis view. Three consecutive loops were recorded under three-lead ECG tracking and transferred to a separate workstation (QLAB cardiovascular ultrasound quantification software, version 11.1, Philips Healthcare, Amsterdam, The Netherlands).

**Figure 1:**
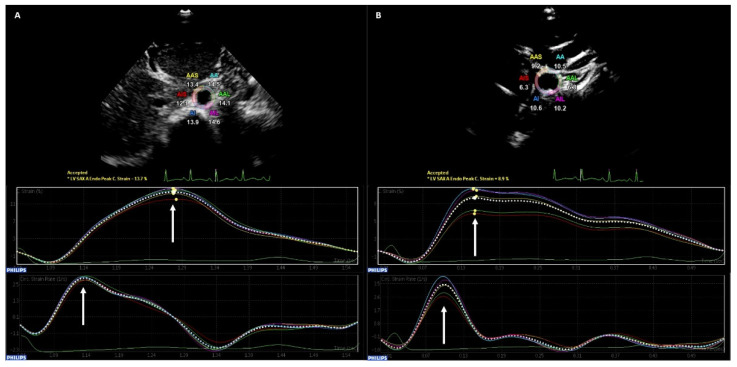
Two-Dimensional Speckle Tracking (2DST) of the Abdominal Aorta and the Common Carotid Arteries. 2DST tracks pixels of the region of interest (ROI) over the cardiac cycle and quantifies their deformation in percent. Special care was taken to set the ROI as small as possible to precisely track the vessel’s wall. Peak circumferential strain (upper graph, marked with the arrow, %) and peak strain rate (lower graph, marked with the arrow, 1/s) were identified manually. (**A**) The abdominal aorta (AAO) was evaluated in short subxiphoid view at epigastric level under three-lead ECG tracking. (**B**) Both common carotid arteries (CCA) were recorded under three-lead ECG tracking in supine position. The neck extended up to a 45°angle and turned to the opposite side of examination.

Offline analysis was performed by one investigator for all study participants. The software’s SAX-A function was used. The smallest region of interest (ROI) was set to precisely track the vessel’s wall. Special care was taken to avoid tracking of the perivascular tissue. The software then tracked pixels of the ROI two-dimensionally over the cardiac cycle and quantified their deformation in percent (Figure 1) [22].

The following parameters were identified manually for AAO and both CCA individually: peak circumferential strain (CS, %), peak strain rate (SR, 1/s) (Figure 1). An average of three measurements was calculated to enhance data validity. 

Arterial distensibility (Dis, mmHg^−1^ × 10^−3^) was defined as follows [30]:

$$\mathrm{Arterial}\;\mathrm{Distensibility}=\frac{\left(2\times \;\mathrm{CS}\right)}{\mathrm{SBP}-\mathrm{DBP}}.$$

SBP and DBP assessed by the Mobil-O-Graph^®^ were utilized for this calculation. Dis was calculated for the AAO and for both CCA individually.

#### 2.6.3. Stiffness Index β

The abovementioned sonographic study protocol was applied. M-Mode of the AAO was performed in short axis view under three-lead ECG tracking using a 3–8 MHz sector or a 1–5 MHz sector array transducer. M-Mode of both CCA was carried out in long axis view under three-lead ECG tracking utilizing a 3–12 MHz linear array transducer.

For all study participants, the end-diastolic diameter (dD, mm) and the end-systolic diameter (sD, mm) of the AAO and both CCA were measured offline (IntelliSpace Cardiovascular Ultrasound Viewer, Philips Healthcare, Amsterdam, The Netherlands) by one investigator.

The stiffness index β (unitless) was calculated for the AAO in short axis and for both CCA in long axis as [7]:

$$\mathrm{Stiffness}\;\mathrm{Index}\;\beta =\frac{\ln\left(\frac{\mathrm{SBP}}{\mathrm{DBP}}\right)}{∆D/\mathrm{dD}}.$$

ΔD (mm) was defined as the difference of sD and dD. SBP and DBP assessed by the Mobil-O-Graph^®^ were utilized for this calculation.

### 2.7. Statistical Analysis

Statistical analysis was performed using SPSS 27 (Release Date 2020, IBM SPSS Statistics for Windows, version 27.0.1.0, IBM Corp., Armonk, NY, USA). The Shapiro–Wilk test, the Kolmogorov–Smirnov test, histograms and QQ-plots were used to test normality of continuous parameters. For continuous and normally distributed variables the unpaired *t*-test was utilized. If continuous data was non-normally distributed the Mann–Whitney U test was applied. Data is given as mean ± SD for normally distributed parameters and as median (interquartile range, IQR) if non-normally distributed. Pearson correlations coefficient was used for normally distributed variables and Spearman correlations coefficient for non-normally distributed variables. For the statistical comparison of correlations, the free cocor software package (http://comparingcorrelations.org/ accessed on 17 October 2022) was used [31]. A *p*-value < 0.05 was considered as statistically significant.

## 3. Results

### 3.1. Patients’ Characteristics

For this study 70 ART and 86 spontaneously conceived peers were recruited. Within the ART cohort, one patient was excluded from further analysis due to history of T-cell lymphoma and one due to history of heart surgery. In addition, one ART subject was excluded due to incomplete data assessment. In total, 67 ART subjects (50 ICSI, 16 IVF, 1 GIFT) and 86 spontaneously conceived peers were included in the final analysis.

Within the ART group, one subject displayed with long QT syndrome, one with a bicuspid aortic valve, one with questionable history of myocarditis, one with hypothyroidism and one with history of hypercholesterolemia. Four ART subjects used oral contraceptives, one was taking L-Thyroxine and one methylphenidate.

Within the control group, six subjects were taking oral contraceptives, one subject was taking bisoprolol due to recurrent migraine episodes and one was taking methylphenidate.

Both groups did not differ significantly in age, gender, bodyweight, body height, BMI and smoking status. Compared to the spontaneously conceived peers, birth weight, birth height and gestational age of ART subjects were significantly lower, whereas the number of multiple pregnancy and maternal age at birth were significantly higher in the ART group. Maternal BMI at conception, presence of gestational diabetes, maternal blood pressure during pregnancy ≥ 140/90 mmHg and maternal educational level did not differ significantly between both groups. No significant difference in adherence to the Mediterranean diet was demonstrated between ART subjects and spontaneously conceived peers. ART adults tended to have a lower adherence to the Mediterranean diet compared to controls. Detailed information on patients’ characteristics is summarized in Table 1.

### 3.2. Vascular Function

When vascular function was analyzed, no significant differences were detected between both groups. Table 2 summarizes data on vascular function for the ART and control group.

### 3.3. Influence of Age on Vascular Function

To assess the influence of age on the cardiovascular parameters studied, a sub-analysis of subjects ≥ 10 years of age was conducted. Moreover, a correlation analysis was performed to assess the influence of age on the cardiovascular parameters studied.

#### 3.3.1. Arterial Stiffness in Children ≥ 10 Years of Age

In total, 38 ART subjects and 55 controls ≥ 10 years of age were included for the sub-analysis. Both groups did not differ significantly in age, gender, bodyweight, body height, BMI and smoking status. Compared to spontaneously conceived peers, birth weight, birth height and gestational age were significantly lower, whereas the number of multiple pregnancies was significantly higher in the ART group. No significant difference in adherence to the Mediterranean Diet was demonstrated between ART subjects and spontaneously conceived peers. ART adults tended to have a lower adherence to the Mediterranean diet compared to controls. Table 3 visualizes data on patients’ characteristics in the ART and control group aged ≥ 10 years.

Within study participants ≥ 10 years of age, vascular parameters did not show significant differences between both groups. SBP displayed a higher tendency in the ART group compared to the control group but did not reach statistical significance. Table 4 summarizes data on vascular function in the ART and control group aged ≥ 10 years.

#### 3.3.2. Correlation Analysis

The influence of age on vascular function was studied for each group by conducting a correlation analysis (Table 5). Observed z-scores (Z_obs_) ranged between −1.189 and 1.529, suggesting no significant differences between the correlations of both groups (Table 5).

## 4. Discussion

67 ART subjects and 86 age- and gender-matched spontaneously conceived peers make this study one of the biggest of its kind. Recently, 2DST has been introduced as a new method to evaluate arterial stiffness [21]. To the best of our knowledge, 2DST has not been applied yet to assess arterial stiffness in the ART offspring. In contrast to previous findings [15,32,33], the present study displayed no significant differences in arterial stiffness, visualized by CS, SR, Dis and stiffness index β of the AAO and the CCA, between ART subjects and spontaneously conceived peers. In addition, oscillometric assessed PWV, SBP and DBP did not differ significantly between both groups.

In the sub-analysis of subjects ≥ 10 years of age, no significant differences in the cardiovascular parameters studied were shown between ART subjects and spontaneously conceived peers.

Moreover, the correlation analysis did not reveal a more pronounced alteration of vascular function with age in the studied ART cohort.

### 4.1. Assisted Reproductive Technologies: A Potential Cardiovascular Risk Factor for the Offspring?

#### 4.1.1. Comparison to Previous Findings

Each year, an increasing number of children are born using ART [34]. With more than 10 million ART children born, their long-term health outcomes deserve special interest [10,34]. This study suggests that vascular function does not differ significantly between ART subjects and spontaneously conceived peers. To evaluate whether vascular aging is more pronounced in the ART offspring, subjects at different ages (children, adolescents, young adults) were intentionally included. Interestingly, when analyzing study participants ≥ 10 years of age, parameters of arterial stiffness remained unchanged between both groups. In addition, no significant differences between the correlations of age and the cardiovascular parameters studied were revealed between both groups. Hence, our study supports recent findings of normal vascular function in ART subjects [19,35].

Halliday et al. demonstrated in a cohort of 193 ART subjects who were matched to 86 spontaneously conceived peers (mean age: 27.5 ± 2.8 years vs. 27.6 ± 2.6 years) no significantly elevated vascular or cardiometabolic risk profile [19]. Outcome measures were parameters of vascular structure, such as carotid artery intima-media thickness, pulse wave velocity and blood pressure [19]. The study conducted by Halliday et al. reinforces the perception that ART does not affect the vascular health of its offspring [19]. These findings are supported by a cohort study including data of 122,429 ART and 7,547,685 naturally conceived children from Sweden, Finland, Norway and Denmark [36]. The study conducted by Norrman et al. could not display a significantly increased risk for CVD (e.g., ischemic heart disease, cardiomyopathy, heart failure, cerebrovascular disease) and type 2 diabetes in ART subjects [36]. However, a significantly elevated risk for obesity was shown for the ART offspring [36]. The large sample size and the prior adjusting for several confounders (e.g., maternal CVD, obesity, diabetes) reinforce the demonstrated results of the current study [36]. However, the short time of follow-up and the relatively young age of the studied populations might underestimate the impact of ART on the offspring’s cardiovascular system as subjects could develop increased cardiovascular morbidity only later in life [36].

In contrast, multiple studies support the hypothesis that the ART offspring might be exposed to greater health risks compared to naturally conceived peers [11,15,18,34,37,38]. Various short-term consequences, including preterm birth, low birth weight and higher perinatal mortality were demonstrated [15,37]. The available data on long-term consequences is sparse, but neurodevelopmental deficits, abnormalities in glucose metabolism and the early development of metabolic syndrome are named as potential adverse health outcomes [11,15,37]. In the literature, cardiovascular remodeling within the ART offspring is assumed [16,18,39]. A well-known Swiss study reported generalized endothelial dysfunction, increased intima-media thickness and elevated arterial stiffness in a cohort of 65 ART singletons compared to 57 spontaneously conceived controls [17,33]. Five years after the initial examination, the premature vascular aging persisted in the ART cohort [32]. A review and meta-analysis by Guo et al. published in 2017, investigated the cardiovascular function of 2112 IVF/ICSI subjects and 4096 spontaneously conceived peers [18]. Interestingly, the authors observed a significantly increased blood pressure, significantly thicker vessel walls and a suboptimal cardiac diastolic function in the ART cohort [18]. The analysis was limited by the number of included studies and the relatively young age of study participants [18].

#### 4.1.2. Pathophysiological Considerations

ART were introduced in 1978 and have made substantial progress in the last 40 years [18,40]. IVF, the primary implemented technique, and ICSI, introduced several years later, are nowadays the most frequently used techniques [15]. ART undergo constant improvements of treatment protocols, culture conditions, quality controls as well as efficiency in fertilization and pregnancy rates leading to higher success rates [18,40,41]. As a result, a decrease in adverse perinatal outcomes including preterm birth and low birth weight was noticed within the ART offspring [42]. Moreover, it was observed that the rate of multiple births dramatically declined over the last decades of using ART [42]. As these factors are known risks for prematurity and increased perinatal mortality, its reduction might be one possible explanation for an overall improvement of the perinatal ART outcome [42]. Additionally, the introduction of cryopreservation as an alternative to fresh embryo transfer might have led to a reduction of prematurity and to an elevation of mean birth weight [43]. Nowadays, ICSI treatments represent approximately 70% of all ART worldwide [10]. Recent studies could not determine a superiority in postfertilization reproductive outcome compared to IVF [44], however, an overall lower preterm birth rate was detected within the ICSI treatment cohort [45]. Interestingly, birth weight, birth height and gestational age were significantly lower in the ART cohort of the current study. Low birth weight and prematurity are associated with several health conditions later in life, such as elevated arterial stiffness and, therefore, an increased risk of CVD development [46,47,48]. Hence, the demonstrated ART improvements and the reduction of associated short-term consequences might contribute to a better long-term ART outcome including lower cardiovascular morbidity and mortality.

The “developmental origins of adult disease” hypothesis is often used to link adverse environmental influences during early human development, such as the ART procedure itself, to increased cardiovascular risk later in life [38]. As the cardiovascular system is one of the first to be matured in the embryonic development, it might be easily affected by adverse environmental influences [34]. During the ART procedure the gametes and embryos are exposed to excessive oxidative stress levels potentially caused by a lack of natural antioxidant systems and other stimuli (e.g., fluctuation in temperature, pH, oxygen concentration) facilitating oxidative stress production [34]. As a result, potential epigenetic modifications in the offspring’s DNA could cause cardiovascular alterations later in life [34]. Within the last years, the embryo culture environment as well as the media composition was further optimized leading to a reduction of oxidative stress levels and DNA methylation [49,50]. Presumably, this optimization could have positively influenced the health outcome of the ART offspring.

Fertility is negatively affected by advanced age and pre-existing CVD risk factors [34,51,52,53]. Animal, human and epidemiological studies demonstrated that advanced maternal age might be associated with lower long-term cardiovascular health of the offspring. Moreover, adult offspring of parents with CVD (e.g., coronary artery disease, cerebrovascular disease, peripheral artery disease) display a higher prevalence of hypercholesteremia and hypertension compared to the general population [14]. Advanced maternal age, chronic hypertension and the use of ART are known risk factors for preeclampsia [54]. Preeclampsia itself, is associated with negative health effects for the fetus leading to increased cardiovascular risk later in life [55]. Presumably, couples with increased age and cardiovascular morbidity might require ART more frequently. As ART procedures undergo continuous development, these techniques might be accessible to an even more morbid cohort in the future [42].

### 4.2. Strengths and Limitations

#### 4.2.1. Study Design

This study was a single center study within Germany. The sample size of this study can be considered as one of the biggest ever reported in the literature. As this study used novel parameters to assess arterial stiffness, a prior power analysis was not feasible. However, a study conducted by Scherrer et al., displayed significant differences in conventional vascular parameters in a cohort of 65 ART subjects and 57 spontaneously conceived peers [33]. Therefore, we aimed for a similar sample size for the present study.

For this study, adverse perinatal conditions (e.g., preterm birth, multiple pregnancy) were purposely not defined as exclusion criteria for study participation. A prior exclusion of such participants was thought to positively influence the “real” cardiovascular risk profile of the studied ART population and to substantially limit the sample size of this single center study. Hence, for a more detailed cardiovascular risk stratification of the ART cohort (e.g., preterm birth, multiple pregnancy, single ART treatments), prospective multi-centric studies with a longitudinal study design and an even greater sample size are required in the future.

ART procedures undergo continuous development [18,40,41]. Hence, the potential impact of new ART methodologies on the offspring’s cardiovascular function needs to be closely monitored in future studies.

GIFT was considered as a conventional form of ART in this study. Compared to IVF or ICSI, GIFT does not imply in vitro cultivation as fertilization occurs in the natural milieu of the fallopian tubes.

As infertility is associated with increased cardiovascular morbidity [17,37], the potential inheritance of parental cardiovascular risk factors needs to be addressed in future ART studies.

In this study, data on pregnancy and birth was assessed retrospectively by screening clinical records and by questioning both parents. However, a loss of information was inevitable in some study participants as clinical records were either “lost” or incompletely filled out by prior healthcare professionals.

The present study did not take psychosocial factors into consideration as relevant cardiovascular risk factors. Seeland et al. suggests vascular ageing to be associated with mood disorders, personality traits and social isolation [13]. 

It is known that certain lifestyle habits might have an impact on vascular function. In the current study, neither smoking nor diet quality did significantly differ between both groups. However, ART adults tended to have a lower adherence to the Mediterranean diet.

#### 4.2.2. Methodology

For 2DST, the use of a sector array transducer was required by the software. Compared to a linear transducer this might have led to a lower image resolution potentially influencing the tracking of speckles.

To the best of our knowledge, 2DST of the AAO and the CCA has not been validated yet. In addition, pediatric reference values for 2DST assessed arterial stiffness parameters do not exist yet. However, multiple studies suggest that 2DST can be a useful tool for the non-invasive assessment of arterial stiffness compared to the current gold standard of carotid–femoral pulse wave velocity [22,56,57]. Moreover, 2DST might facilitate the regional evaluation of central arterial stiffness compared to conventional methods that assess “global” arterial stiffness peripherally [58]. Nonetheless, it needs to be pointed out that a regional estimation of arterial stiffness might be accompanied by lower accuracy compared to a global assessment [58]. Therefore, this study assessed arterial stiffness additionally by measuring PWV via an oscillometric device. In addition, 2DST tended to overestimate strain values in an experimental study [58].

Further, the potential influence of extrinsic mechanical factors (e.g., chest wall conformation) on arterial elastance and ventricular–arterial coupling needs to be addressed and investigated in further studies [59,60].

As previously shown, 2DST of the AAO displays a relatively high intra-and interobserver variability [61]. To reduce intraobserver variability in this study, an average of three measurements was calculated to enhance data validity. To minimize interobserver variability, one investigator conducted acquisition and offline analysis of the sonographic images. As the quality of 2DST is highly dependent on the sonographic window, imprecise tracking of speckles might be present in subjects with excess weight [61].

## 5. Conclusions

The present study displayed no significant differences in arterial stiffness between ART subjects and spontaneously conceived peers, visualized by CS, SR, Dis and stiffness index β of the AAO and the CCA as well as PWV. In addition, the results of this study suggest that arterial stiffness does not elevate more profoundly in ART subjects with increasing age compared to spontaneously conceived peers. In the future, prospective multi-centric studies with a longitudinal study design and an even greater sample size are required for a more detailed cardiovascular risk stratification of the ART cohort.

## Figures and Tables

**Table 1:** Patients’ Characteristics in the ART and Control Group.

Variable	ART (n = 67)	Control (n = 86)	*p*-Value
Age (years)	11.31 (8.10–18.20)	11.85 (8.72–18.27)	0.43
Female (n (%))	39 (58.2)	44 (51.2)	0.39
Bodyweight (kg)	38.70 (23.40–58.80)	42.70 (29.05–59.25)	0.25
Body height (cm)	145.00 (125.00–167.00)	157.00 (133.38–170.25)	0.11
BMI (kg/m^2^)	16.79 (15.09–20.83)	17.66 (15.43–21.05)	0.49
Weight classification			1
*Underweight (n (%))*	4 (6.0)	6 (7.0)	
*Normal weight (n (%))*	58 (86.6)	74 (86.0)	
*Overweight (n (%))*	5 (7.5)	6 (7)	
Smoking (n (%))	3 (4.5)	2 (2.3)	0.65
**Course of Pregnancy and Birth, Maternal Educational Level**			
Birth weight (g) ^1^	2990 (2405–3243)	3440 (3210–3670)	<0.001 ***
Birth height (cm) ^2^	50 (48–52)	52 (50–54)	<0.001 ***
Gestational age (weeks) ^3^	38 (36–40)	39 (38–40)	<0.001 ***
Multiple pregnancy (n (%))	21 (31.3)	2 (2.3)	<0.001 ***
Maternal age at birth (years) ^4^	35.37 ± 3.72	33.07 ± 4.10	<0.001 ***
Maternal BMI at conception (kg/m^2^) ^5^	22.49 (20.55–24.65)	21.38 (20.24–22.72)	0.09
Gestational diabetes (n (%)) ^6^	3 (5.3)	3 (4.2)	1
Maternal blood pressure during pregnancy ≥ 140/90 mmHg (n (%)) ^7^	0 (0)	3 (6.4)	0.29
Maternal educational level ^8^	4 (3–5)	5 (4–5)	0.22
**Diet Quality**			
MEDAS ^9^	5.94 ± 2.51	7.27 ± 1.67	0.05
KIDMED ^10^	6.24 ± 2.33	6.92 ± 2.11	0.11

ART = assisted reproductive technologies; BMI = body mass index. ^1^ 65 ART subjects and 79 control subjects were included in the analysis. ^2^ 63 ART subjects and 79 control subjects were included in the analysis. ^3^ 62 ART subjects and 77 control subjects were included in the analysis. ^4^ 66 ART subjects were included in the analysis. ^5^ 47 ART subjects and 61 control subjects were included in the analysis. ^6^ 57 ART subjects and 71 control subjects were included in the analysis. ^7^ 28 ART subjects and 47 control subjects were included in the analysis. ^8^ 45 ART subjects and 52 control subjects were included in the analysis. ^9^ 17 adult ART subjects and 22 adult control subjects were included in the analysis. ^10^ 50 minor ART subjects and 64 minor control subjects were included in the analysis. Maternal educational level was assessed according to the German educational system: no school leaving qualification (0), lower secondary school leaving certificate (1), intermediate secondary school leaving certificate (2), general qualification for university entrance (3), completed apprenticeship (4), completed university degree (5). Data is presented as mean ± SD for normally distributed parameters and as median (IQR) for non-normally distributed parameters. Nominal data is presented as n (%). *** *p* ≤ 0.001.

**Table 2:** Vascular Function in the ART and Control Group.

Variable	ART (n = 67)	Control (n = 86)	*p*-Value
SBP (mmHg)	113.64 ± 12.02	113.24 ± 8.96	0.82
DPB (mmHg)	65.00 (59.00–72.00)	63.50 (59.00–71.25)	0.96
PWV (m/s)	4.63 ± 0.55	4.62 ± 0.41	0.88
AAO CS (%) ^1^	22.46 ± 6.74	22.42 ± 8.26	0.97
AAO SR (1/s) ^2^	4.15 ± 0.98	4.14 ± 1.20	0.94
AAO Dis (mmHg^−1^ × 10^−3^) ^1^	950 ± 325	969 ± 398	0.75
AAO β index ^3^	2.47 (1.72–3.72)	2.54 (1.85–3.69)	0.61
rCCA CS (%) ^4^	13.71 ± 3.41	14.60 ± 4.68	0.18
rCCA SR (1/s) ^4^	3.82 (3.38–4.50)	4.10 (3.23–4.93)	0.13
rCCA Dis (mmHg^−1^ × 10^−3^) ^4^	580 ± 177	628 ± 222	0.16
rCCA β index long axis ^5^	3.87 (2.90–5.31)	3.69 (2.62–5.58)	0.54
lCCA CS (%) ^4^	14.73 (12.10–17.67)	14.88 (11.08–17.82)	0.67
lCCA SR (1/s) ^4^	4.04 ± 1.02	4.00 ± 1.15	0.82
lCCA Dis (mmHg^−1^ × 10^−3^) ^4^	633 (450–727)	599 (448–745)	0.80
lCCA β index long axis ^6^	3.26 (2.33–4.32)	3.06 (2.57–4.08)	0.74

ART = assisted reproductive technologies; SBP = systolic blood pressure; DBP = diastolic blood pressure; PWV = pulse wave velocity, AAO = abdominal aorta; CS = peak circumferential strain; SR = peak strain rate; Dis = arterial distensibility; rCCA = right common carotid artery; lCCA = left common carotid artery. ^1^ 63 ART subjects and 83 control subjects were included in the analysis. ^2^ 64 ART subjects and 83 control subjects were included in the analysis. ^3^ 62 ART subjects and 83 control subjects were included in the analysis. ^4^ 64 ART subjects were included in the analysis. ^5^ 66 ART subjects were included in the analysis. ^6^ 66 ART subjects and 85 control subjects were included in the analysis. Data is presented as mean ± SD for normally distributed parameters and as median (IQR) for non-normally distributed parameters.

**Table 3:** Patients’ Characteristics in the ART and Control Group Aged ≥ 10 Years.

Variable	ART (n = 38)	Control (n = 55)	*p*-Value
Age (years)	17.06 (12.40–20.66)	16.15 (11.99–21.75)	0.99
Female (n (%))	21 (55.3)	28 (50.9)	0.68
Bodyweight (kg)	55.24 ± 15.19	56.05 ± 13.96	0.79
Body height (cm)	162.77 ± 13.25	166.94 ± 13.10	0.14
BMI (kg/m^2^)	20.44 ± 3.28	19.81 ± 3.11	0.35
Weight classification			0.73
*Underweight (n (%))*	3 (7.9)	6 (10.9)	
*Normal weight (n (%))*	30 (78.9)	45 (81.8)	
*Overweight (n (%))*	5 (13.2)	4 (7.3)	
Smoking (n (%))	3 (7.9)	1 (1.8)	0.16
**Course of Pregnancy and Birth, Maternal Educational Level**			
Birth weight (g) ^1^	2995 (2218–3197.50)	3425 (3170–3671.25)	<0.001 ***
Birth height (cm) ^2^	50 (48–52)	51 (50–54)	0.004 **
Gestational age (weeks) ^3^	38 (34–39)	39 (38–40)	0.012 *
Multiple pregnancy (n (%))	13 (34.2)	2 (3.6)	<0.001 ***
Maternal age at birth (years) ^4^	34.03 ± 3.42	32.94 ± 4.20	0.19
Maternal BMI at conception (kg/m^2^) ^5^	22.49 (20.55–25.26)	21.23 (19.89–23.21)	0.38
Gestational diabetes (n (%)) ^6^	1 (3.2)	0 (0)	0.43
Maternal blood pressure during pregnancy ≥ 140/90 mmHg (n (%)) ^7^	0 (0)	1 (4.2)	1
Maternal educational level ^8^	4 (2–5)	5 (4–5)	0.14
**Diet Quality**			
MEDAS ^9^	5.94 ± 2.51	7.27 ± 1.67	0.05
KIDMED ^10^	5.76 ± 2.45	6.27 ± 2.10	0.42

ART = assisted reproductive technologies; BMI = body mass index. ^1^ 36 ART subjects and 48 con-trol subjects were included in the analysis. ^2^ 34 ART subjects and 48 control subjects were included in the analysis. ^3^ 35 ART subjects and 47 control subjects were included in the analysis. ^4^ 37 ART subjects were included in the analysis. ^5^ 23 ART subjects and 34 control subjects were included in the analysis. ^6^ 31 ART subjects and 41 control subjects were included in the analysis. ^7^ 12 ART subjects and 24 control subjects were included in the analysis. ^8^ 25 ART subjects and 38 control subjects were included in the analysis. ^9^ 17 adult ART subjects and 22 adult control subjects were included in the analysis. ^10^ 21 minor ART subjects and 33 minor control subjects were included in the analysis. Maternal educational level was assessed according to the German educational system: no school leaving qualification (0), lower secondary school leaving certificate (1), intermediate secondary school leaving certificate (2), general qualification for university entrance (3), completed apprenticeship (4), completed university degree (5). Data is presented as mean ± SD for normally distributed parameters and as median (IQR) for non-normally distributed parameters. Nominal data is presented as n (%). * *p* < 0.05. ** *p* ≤ 0.01. *** *p* ≤ 0.001.

**Table 4:** Vascular Function in the ART and Control Group Aged ≥ 10 Years.

Variable	ART (n = 38)	Control (n = 55)	*p*-Value
SBP (mmHg)	120.18 ± 9.57	116.55 ± 8.05	0.05
DPB (mmHg)	69.79 ± 7.63	67.96 ± 7.95	0.27
PWV (m/s)	4.87 ± 0.41	4.80 ± 0.38	0.41
AAO CS (%) ^1^	22.05 ± 7.39	21.87 ± 7.54	0.91
AAO SR (1/s) ^1^	4.22 ± 1.04	4.21 ± 1.14	0.98
AAO Dis (mmHg^−1^ × 10^−3^) ^1^	895 ± 338	920 ± 346	0.73
AAO β index ^2^	2.74 (1.85–3.89)	2.44 (1.88–3.53)	0.65
rCCA CS (%)	13.39 ± 3.16	13.64 ± 3.82	0.74
rCCA SR (1/s)	3.92 ± 0.86	3.91 ± 0.96	0.98
rCCA Dis (mmHg^−1^ × 10^−3^)	540 ± 143	572 ± 179	0.36
rCCA β index long axis	3.93 (2.90–5.53)	4.09 (2.71–5.64)	0.86
lCCA CS (%) ^3^	14.14 ± 3.97	13.74 ± 4.19	0.64
lCCA SR (1/s) ^3^	3.84 ± 1.07	3.72 ± 1.02	0.57
lCCA Dis (mmHg^−1^ × 10^−3^) ^3^	574 ± 183	569 ± 171	0.90
lCCA β index long axis ^1^	3.54 (2.56–4.76)	3.37 (2.69–4.40)	0.70

ART = assisted reproductive technologies; SBP = systolic blood pressure; DBP = diastolic blood pressure; AAO = abdominal aorta; CS = peak circumferential strain; SR = peak strain rate; Dis = arterial distensibility; rCCA = right common carotid artery; lCCA = left common carotid artery. ^1^ 54 control subjects were included in the analysis. ^2^ 37 ART subjects and 52 control subjects were included in the analysis. ^3^ 37 ART subjects and 55 control subjects were included in the analysis. Data is presented as mean ± SD for normally distributed parameters and as median (IQR) for non-normally distributed parameters.

**Table 5:** Correlation Analysis between Age and Vascular Function in the ART and Control Group.

Variable	ART (n = 67)		Control (n = 86)		Z_obs_
	r	*p*-Value	r	*p*-Value	
SBP (mmHg)	0.690	<0.001 ***	0.633	<0.001 ***	0.610
DBP (mmHg)	0.642	<0.001 ***	0.510	<0.001 ***	1.195
PWV (m/s)	0.742	<0.001 ***	0.807	<0.001 ***	−0.983
AAO CS (%) ^1^	−0.211	0.098	−0.131	0.238	−0.483
AAO SR (1/s) ^2^	0.099	0.437	0.056	0.616	0.255
AAO Dis (mmHg^−1^ × 10^−3^) ^1^	−0.368	0.003 **	−0.181	0.102	−1.189
AAO β index ^3^	0.232	0.07	−0.026	0.816	1.529
rCCA CS (%) ^4^	−0.159	0.209	−0.243	0.024 *	0.519
rCCA SR (1/s) ^4^	−0.020	0.874	−0.257	0.017 *	1.440
rCCA Dis (mmHg^−1^ × 10^−3^) ^4^	−0.303	0.015 *	−0.263	0.014 *	−0.258
rCCA β index long axis ^5^	0.114	0.361	0.256	0.018	−0.882
lCCA CS (%) ^4^	−0.276	0.027 *	−0.308	0.004 **	0.207
lCCA SR (1/s) ^4^	−0.256	0.041 *	−0.371	<0.001 ***	0.758
lCCA Dis (mmHg^−1^ × 10^−3^) ^4^	−0.374	0.002 **	−0.407	<0.001 ***	0.231
lCCA β index long axis ^6^	0.132	0.292	0.208	0.056	−0.467

ART = assisted reproductive technologies; Z_obs_ = observed z-score; SBP = systolic blood pressure; DBP = diastolic blood pressure; PWV = pulse wave velocity, AAO = abdominal aorta; CS = peak circumferential strain; SR = peak strain rate; Dis = arterial distensibility; rCCA = right common carotid artery; lCCA = left common carotid artery. ^1^ 63 ART subjects and 83 control subjects were included in the analysis. ^2^ 64 ART subjects and 83 control subjects were included in the analysis. ^3^ 62 ART subjects and 83 control subjects were included in the analysis. ^4^ 64 ART subjects were included in the analysis. ^5^ 66 ART subjects were included in the analysis. ^6^ 66 ART subjects and 85 control subjects were included in the analysis. Data is presented as mean ± SD for normally distributed parameters and as median (IQR) for non-normally distributed parameters. * *p* < 0.05. ** *p* ≤ 0.01. *** *p* ≤ 0.001.

## Data Availability

Not applicable.
